# Identification and Characterization of New Bacteriophages to Control Multidrug-Resistant *Pseudomonas aeruginosa* Biofilm on Endotracheal Tubes

**DOI:** 10.3389/fmicb.2020.580779

**Published:** 2020-10-06

**Authors:** Viviane C. Oliveira, Felipe L. Bim, Rachel M. Monteiro, Ana Paula Macedo, Emerson S. Santos, Cláudia H. Silva-Lovato, Helena F. O. Paranhos, Luís D. R. Melo, Sílvio B. Santos, Evandro Watanabe

**Affiliations:** ^1^Human Exposome and Infectious Diseases Network, School of Nursing of Ribeirão Preto, University of São Paulo, Ribeirão Preto, Brazil; ^2^Department of Dental Materials and Prostheses, School of Dentistry of Ribeirão Preto, University of São Paulo, Ribeirão Preto, Brazil; ^3^Department of Clinical Toxicological and Bromatologic Analysis, School of Pharmaceutical Sciences of Ribeirão Preto, University of São Paulo, Ribeirão Preto, Brazil; ^4^Centre of Biological Engineering, University of Minho, Braga, Portugal; ^5^Department of Restorative Dentistry, School of Dentistry of Ribeirão Preto, University of São Paulo, Ribeirão Preto, Brazil

**Keywords:** bacteriophage, biofilm, *Pseudomonas aeruginosa*, multidrug resistance, phage therapy

## Abstract

Studies involving antimicrobial-coated endotracheal tubes are scarce, and new approaches to control multidrug-resistant *Pseudomonas aeruginosa* biofilm on these devices should be investigated. In this study, five new *P. aeruginosa* bacteriophages from domestic sewage were isolated. All of them belong to the order *Caudovirales*, *Myoviridae* family. They are pH and heat stable and produce 27 to 46 particles after a latent period of 30 min at 37°C. Their dsDNA genome (ranging from ∼62 to ∼65 kb) encodes 65 to 89 different putative proteins. They exhibit a broad lytic spectrum and infect 69.7% of the *P. aeruginosa* strains tested. All the bacteriophages were able to reduce the growth of *P. aeruginosa* strains in planktonic form. The bacteriophages were also able to reduce the biofilm viability rates and the metabolic activity of *P. aeruginosa* strains in a model of biofilms associated with endotracheal tubes. In addition, scanning electron microscopy micrographs showed disrupted biofilms and cell debris after treatment of bacteriophages, revealing remarkable biofilm reduction. The lytic activity on multidrug-resistant *P. aeruginosa* biofilm indicates that the isolated bacteriophages might be considered as good candidates for therapeutic studies and for the application of bacteriophage-encoded products.

## Introduction

Mechanical ventilation through an endotracheal or tracheostomy tube is a supportive intervention to critically ill patients and represents a risk factor for the occurrence of ventilator-associated pneumonia (VAP; Haas et al., 2014). VAP is the most common healthcare-associated infection in critically ill patients, involves high financial costs, and has negative prognosis (Barbier et al., 2013; Zimlichman et al., 2013; Russotto et al., 2015). VAP etiologies have been widely discussed, and the factors associated with its development include endotracheal tube biofilm formation which plays an important role as a reservoir for microorganisms (Nseir et al., 2011).

Knowing the potential risk factor of multidrug-resistant *Pseudomonas aeruginosa* biofilms on endotracheal tubes, interest in the development of antimicrobial-coated endotracheal tubes has emerged. The presence of antimicrobial agents, such as silver particles and chlorhexidine, on the surface of endotracheal tubes demonstrated a decrease in biofilm formation on the device surface’s (Berra et al., 2004; Tokmaji et al., 2015). However, chemical compounds have been implicated in bacterial resistance (Cieplik et al., 2019) and frequently linked to potential cytotoxic hazards (Lessa et al., 2010; Mao et al., 2018). Therefore, new approaches to control multidrug-resistant *P. aeruginosa* biofilm on endotracheal tubes should be investigated.

The applicability of bacteriophages in medical devices has aroused interest in several areas, including infections related to the urinary tract (Nzakizwanayo et al., 2015; Melo et al., 2016), venous catheters (Lungren et al., 2014), and orthopedic prostheses (Kaur et al., 2014; Barros et al., 2020). Recently, new lytic bacteriophages able to control multidrug-resistant *P. aeruginosa* planktonic cells and associated-biofilm forms were described (Yuan et al., 2019; Adnan et al., 2020). Although promising, there are few reports on bacteriophage applicability to control biofilms associated with endotracheal tubes (Aleshkin et al., 2016). Thus, we consider it essential to clarify whether newly isolated bacteriophages can be applied in order to control *P. aeruginosa* biofilms on the surface of endotracheal tubes. The null hypothesis of this study is that there would not be a difference in *P. aeruginosa* growth when challenged with bacteriophages.

## Materials and Methods

### Bacteriophage Isolation and Propagation

Bacteriophages were isolated from domestic sewage, and *P. aeruginosa* (ATCC 27853) was used as the host strain. Isolation, sample enrichment, and purification methods followed the protocol described previously (Sillankorva et al., 2008). Briefly, 20 ml of domestic effluents, collected from March to November 2018 (Domestic Sewage Treatment Station, Ribeirão Preto, Brazil and Araxá, Brazil), was mixed to 20 ml of double-strength tryptic soy broth (TSB; BD Difco, Sparks, MN, United States) and 100 μl of exponentially grown *P. aeruginosa*. The presence of bacteriophage was confirmed by inhibition haloes on double-layer agar plating [Tryptic Soy Agar Soft (0.8% agar)—TSAS (BD Difco)] (Sambrook and Russell, 2001). Repeated double-layer agar plating was done until single-plaque morphology was observed. For propagation, one isolated plaque was collected through a sterile toothpick and diluted in 25 μl of elution buffer [1 M Tris–HCl, pH 7.5 (Sigma Aldrich, Saint Louis, MO, United States), 10 mM MgSO_4_ (Sigma Aldrich), 10 mM NaCl (Dinâmica, Indaiatuba, SP, Brazil), 0.002% (w/v) gelatin (Dinâmica)]. The suspension was dropped onto a TSAS medium, with *P. aeruginosa* lawns, and incubated at 37°C for 24 h. Afterward, 10 ml of elution buffer was added to each plate to elute the bacteriophages, which were purified with chloroform (Sigma Aldrich), titrated, and stored at 4°C.

### Transmission Electron Microscopy

One milliliter of bacteriophage suspension was centrifuged at 21,800 *g* for 60 min at 4°C. The sediment was washed twice in sterile ultrapure water, and 25 μl of each bacteriophage was applied on an electron microscopy grid. The grids were stained with 2% uranyl acetate for 5 min and then analyzed by transmission electron microscopy (JEOL JEM-100 CXII Akishima, Tokyo, Japan; Melo et al., 2014).

### Acid–Base and Thermal Stability

In order to assess acid–base stability, 100 μl of the bacteriophage suspension (10^8^ plaque forming units per milliliter—PFU/ml) were added to 900 μl of elution buffer, with pH adjusted to 3.0, 5.0, 7.0, 9.0, and 11.0. The samples were incubated for 2 h at 37°C. To evaluate thermal stability, 100 μl of the bacteriophage suspension (10^8^ PFU/ml) was added to 900 μl of elution buffer and incubated at different temperatures (37, 42, 50, 60, 70, and 80°C) for 2 h (20). After the incubation period, the phage titer was determined through the double-layer agar method (Cui et al., 2017). The assays were carried out at three independent times in triplicate.

### One-Step Growth Curve

An exponential-phase *P. aeruginosa* culture was centrifuged at 4,200 *g* for 5 min, and OD_600_ was adjusted to achieve ∼10^11^ colony-forming units per milliliter (CFU/ml). Subsequently, 5 ml of bacteria suspension was added to 5 ml of bacteriophage suspension at 5 × 10^8^ PFU/ml (multiplicity of infection—MOI: 0.005). The mixture was incubated (120 rpm, 37°C, 5 min) and centrifuged (4,200 *g*, 5 min), and 10 ml of sterile TSB was added to the pellet. Samples were incubated (120 rpm, 37°C), and aliquots were taken every 5 min until 60 min post-infection. Using the double-layer agar plating, the number of bacteriophage particles was determined at each time. Latent period was determined as the time required for the phage to increase in number and burst size as the ratio between the number of bacteriophages after reaching the plateau and the number of infected bacteria (Sillankorva et al., 2008). The assay was carried out at three independent times.

### Genome Sequencing Analysis

One milliliter of bacteriophage stock was incubated with 10 U DNAse I (Sigma Aldrich) and 10 μl RNAse A (20 mg/ml; Sigma Aldrich) at 37°C for 1 h. Enzymes were inactivated at 70°C for 15 min. Then, the suspensions were incubated with extraction buffer [40 μl EDTA 0.5 M (Sigma Aldrich), 5 μl proteinase K 10 mg/ml (Sigma Aldrich), and 50 μl SDS 10% (w/v; Sigma Aldrich)]. Extraction was performed with phenol/chloroform/isoamyl alcohol (25:24:1; Sigma Aldrich). Absence of bacterial contaminant DNA was confirmed by amplification of the 16S rRNA *P. aeruginosa* gene (Spilker et al., 2004).

Libraries were prepared using the NexteraXT kit (Illumina, San Diego, CA, United States), and genomes were sequenced using Illumina MiSeq paired-end sequencing (2 × 300 bp/coverage 100×), according to the manufacturer’s instructions. Reads were trimmed and assembled into a single contig using Velvet *de novo* tool (Illumina). Potential coding sequences (CDSs) were initially annotated using myRAST (Aziz et al., 2008), and alternative start codons or non-annotated CDSs were further analyzed using Geneious version 2019.2.1 (Biomatters Ltd., Auckland, New Zealand). Encoded proteins were queried against the National Center for Biotechnology Information (NCBI) non-redundant database with BLASTP (coverage > 80%; *E*-value ≤ 10^–5^; Altschul et al., 1990). Protein families, signatures, and structure prediction were searched with PFAM (Finn et al., 2014), InterPro (Finn et al., 2017), and HHpred (Söding, 2005), while the presence of transmembrane domains was checked using TMHMM (Käll and Sonnhammer, 2002), and Phobius (Käll et al., 2004). To predict signal peptide cleavage sites, SignalP (Petersen et al., 2011), and Spoctopus (Viklund et al., 2008) were used. Transfer RNAs (tRNAs) were scanned using tRNAscan-SE (Schattner et al., 2005).

### Host Range Investigation and EOP Analysis

Host range was evaluated by the presence of clear or turbid inhibition haloes in a spot test on double-layer agar against 26 clinical isolates and seven multidrug-resistant *P. aeruginosa* strains (Panel ATCC^®^ MP-23^TM^; Table 1). The susceptibility profile of clinical isolates was determined by the disk diffusion method. Susceptibility test, antimicrobial classes, antibiotic concentration, and zone diameter breakpoint for *P. aeruginosa* strains were defined according to the Clinical and Laboratory Standards Institute (Clinical and Laboratory Standards Institute [CLSI], 2019).

**TABLE 1:** Lytic spectrum and efficiency of plating of the bacteriophages against sensible and multidrug-resistant *Pseudomonas aeruginosa* isolated from different sources.

Isolates	Source	Antibiotic resistance^a^	vB_PaeM_USP_1		vB_PaeM_USP_2		vB_PaeM_USP_3		vB_PaeM_USP_18		vB_PaeM_USP_25	
			Infectivity	EOP^b^	Infectivity	EOP^b^	Infectivity	EOP^b^	Infectivity	EOP^b^	Infectivity	EOP^b^
Sa_ATCC 6538			−		−		−		−		−	
Ec_ATCC 25927			−		−		−		−		−	
Ef_ATCC 29212			−		−		−		−		−	
Pa_ATCC 27853	Blood	S	+		+		+		+		+	
Pa_ATCC 2108^c^	Sputum	AMK, CFZ, CTX, GEN, IMP, TGC	+	High	+	High	+	High	+	Inefficient	+	Inefficient
Pa_ATCC 2109^c^	Sputum	AMK, CFZ, TGC	−		−		−		−		−	
Pa_ATCC 2110^c^	Sputum	AMP, CFZ, CTX, FOX, NIT, TGC, SXT	+	Low	+	High	+	High	+	Low	+	Low
Pa_ATCC 2111^c^	Sputum	AMC, AMP, CFZ, CPD, CRO, CTX, CXM, FOX, NIT, TET, TGC, SXT	−		−		−		−		−	
Pa_ATCC 2112^c^	Sputum	AMC, AMP, CFZ, CPD, CRO, CTX, CXM, FOX, NIT, TET, TGC, SXT	+	High	+	High	+	High	+	Low	+	Low
Pa_ATCC 2113^c^	Sputum	AMP, AMC, CFZ, CTX, NIT, SAM, SXT	+	High	+	High	+	Low	+	Inefficient	+	Inefficient
Pa_ATCC 2114^c^	Sputum	AMP, AMC, CFZ, CTX, NIT, SAM, TZP, SXT	−		−		−		−		−	
Pa_Ba_3	Prosthetic biofilm	S	−		−		−		−		−	
Pa_Ba_129	Prosthetic biofilm	S	+	Inefficient	+	Inefficient	+	Inefficient	+	Inefficient	+	Inefficient
Pa_Ba_130	Prosthetic biofilm	S	+	Inefficient	+	Inefficient	+	Inefficient	+	Inefficient	+	Inefficient
Pa_Ba_164	Prosthetic biofilm	S	+	High	+	High	+	High	+	High	+	High
Pa_Ba_168	Prosthetic biofilm	S	+	Low	+	High	+	High	+	High	+	High
Pa_Ba_169	Prosthetic biofilm	S	+	High	+	High	+	High	+	High	+	High
Pa_Ba_286	Prosthetic biofilm	S	+	Inefficient	+	Inefficient	+	Inefficient	+	Inefficient	+	Inefficient
Pa_Fe_39	Saliva	S	−		−		−		−		−	
Pa_Mi_1	Blood	S	+	Low	+	Low	+	Low	+	Low	+	Low
Pa_Mi_2	Sputum	S	+	High	+	Low	+	High	+	High	+	Low
Pa_Mi_3^c^	Urine	CIP, GEN, IMP, LVX	+	Inefficient	+	Inefficient	+	Inefficient	+	Inefficient	+	Inefficient
Pa_Mi_4	Urine	S	−		−		−		−		−	
Pa_Mi_5	Sputum	S	+	Inefficient	+	Inefficient	+	Inefficient	+	Inefficient	+	Inefficient
Pa_Mi_6	Urine	S	+	High	+	Low	+	Low	+	High	+	High
Pa_Mi_7	Sputum	S	+	Low	+	High	+	High	+	High	+	Low
Pa_Trac_3	Tracheal secretion	S	−		−		−		−		−	
Pa_Trac_4	Tracheal secretion	S	−		−		−		−		−	
Pa_Trac_5^c^	Tracheal secretion	AMK, FEP, CIP, GEN, IMP, LVX, MEM	−		−		−		−		−	
Pa_Trac_14^c^	Tracheal secretion	AMK, CIP, IMP, LVX, MEM	+	High	+	High	+	Inefficient	+	Inefficient	+	Inefficient
Pa_Trac_20	Tracheal secretion	S	+	High	+	High	+	High	+	High	+	High
Pa_Trac_22	Tracheal secretion	S	+	Inefficient	+	Inefficient	+	Inefficient	+	Inefficient	+	Inefficient
Pa_Trac_23	Tracheal secretion	S	+	High	+	High	+	High	+	Low	+	Low
Pa_Trac_27^c^	Tracheal secretion	AMK, CIP, GEN, IMP, LVX, MEM	+	High	+	High	+	High	+	Inefficient	+	Inefficient
Pa_Trac_31	Tracheal secretion	S	−		−		−		−		−	
Pa_Ren_1	Saliva	S	+	High	+	High	+	High	+	High	+	High
Pa_Gus_B2	Blood	S	+	High	+	High	+	High	+	Inefficient	+	Inefficient

*^*a*^AMC, amoxicillin-clavulanic acid; AMK, amikacin; AMP, ampicillin; CFZ, cefazolin; CIP, ciprofloxacin; CPD, cefpodoxime; CRO, ceftriaxone; CTX, cefotaxime; CXM, cefuroxime; FEP, cefepime; FOX, cefoxitin; GEN, gentamicin; IMP, imipenem; LVX, levofloxacin; MEM, meropenem; NIT, nitrofurantoin; SAM, ampicillin-sulbactam; SXT, trimethoprim-sulfamethoxazole; TET, tetracycline; TGC, tigecycline; and TZP, piperacillin-tazobactam. ^*b*^EOP (efficiency of plating) was recorded as high, low, and inefficient, representing >1, 0.1–1, and <0.1%, respectively. ^*c*^MDR, multidrug-resistant (defined as acquired non-susceptibility to at least one agent in three or more antimicrobial categories; Magiorakos et al., 2012). S, susceptible to all antimicrobial agents tested.*

Efficiency of plating (EOP) was determined for all *P. aeruginosa* strains positive for bacteriophage lytic activity. Tenfold dilutions of the bacteriophages were spotted on double-layer agar inoculated with *P. aeruginosa* strains. EOP values were calculated as the ratio between the lysis plaques produced in each susceptible strain and the number of plaques produced in *P. aeruginosa* ATCC 27853. EOP was recorded as high, low, and inefficient, representing >1, 0.1–1, and <0.1%, respectively (Melo et al., 2014).

### Infection of Planktonic Cells

Ten clinical isolates with the highest EOP values, *P. aeruginosa* ATCC 27853, and multidrug-resistant *P. aeruginosa* ATCC 2108, ATCC 2110, ATCC 2112, and ATCC 2113 were selected for this assay. Then, 100 μl of *P. aeruginosa* strains, OD_600_ adjusted to achieve ∼10^9^ CFU/ml, was inoculated with 100 μl of each bacteriophage at a concentration of 5 × 10^7^ PFU/ml (MOI: 0.05). The positive and negative control experiments were performed using a dilution buffer instead of bacteriophages and sterile TSB instead of bacterial suspension, respectively. The plates were incubated at 37°C, 80 rpm. The OD_600_ was measured after 2, 4, 6, 8, and 24 h (Melo et al., 2014). The assays were carried out at three independent times in triplicate.

### Antibiofilm Activity Measurement

The same 15 *P. aeruginosa* strains applied in the infection of planktonic cells test were selected for this assay. The anti-biofilm activity of the bacteriophages was determined by means of biofilm viability rates (CFU/cm^2^), metabolic activity of the bacteria (XTT), and scanning electron microscopy (SEM). The assays were carried out at three independent times in triplicate.

For biofilm infection, strains were grown in TSB medium (37°C, 24 h), and OD_600_ was adjusted to ∼10^9^ CFU/ml. Circular endotracheal tube specimens (Ø 5 mm; Rüsh, Meridian, MS, United States) were sterilized by hydrogen peroxide plasma (Sterrad 100S, Irvine, CA, United States) and randomly distributed in 48-well tissue culture plates. Each well received 400 μl of TSB medium containing the standardized cell suspension (∼10^7^ CFU/ml). Negative control was performed using sterile TSB. The plates were incubated for 48 h at 37°C and 80 rpm. Afterward, the specimens were removed carefully from the plate, rinsed in 10 ml of phosphate-buffered saline (PBS), and transferred to a new plate with 1 ml of sterile culture medium and 10^8^ PFU/ml of bacteriophages. The control samples received culture medium without bacteriophages. The plates were incubated for 24 h at 37°C and 80 rpm. After the incubation time, the specimens were removed from the wells through sterilized tweezers and rinsed in 10 ml of PBS.

For CFU quantification, the specimens were transferred to a tube containing 2 ml of PBS. In order to detach the remaining biofilm, the tubes were vortexed for 30 s, sonicated (200 W, 40 kHz; Altsonic, Clean 9CA, Ribeirão Preto, SP, Brazil) for 20 min, and vortexed again for 2 min. Afterward, 10-fold-dilution aliquots (10^0^–10^–6^) were seeded in Petri dishes containing TSA medium. The Petri dishes were incubated at 37°C for 24 h, and the number of colonies was registered and expressed as log CFU/cm^2^.

For the evaluation of metabolic activity, the specimens were transferred to another 48-well plate containing 316 μl PBS supplemented with 100 mM glucose (Sigma Aldrich), 80 μl XTT 1 mg/ml (Sigma Aldrich), and 4 μl of 0.4 mM menadione (Sigma Aldrich). The plates were statically incubated, protected from light, at 37°C for 2 h, and the absorbance of the resulting solution was measured (492 nm).

For SEM, *P. aeruginosa* ATCC 27853 was analyzed. The biofilms were removed from the plates and washed twice in distilled water. Samples were fixed with 2.5% glutaraldehyde for 60 min and then dehydrated in a graded ethanol series (30, 50, 70, 90, and 100%). After chemical drying (hexamethyldisilazane; Sigma Aldrich), the specimens were mounted on a metal stub and gold-coated. The surface morphology of the biofilms was examined under high vacuum with a JEOL JSM-35CF microscope (Tokyo, Japan).

### Statistical Analysis

Statistical tests were performed through the IBM SPSS Statistics 25.0 software (IBM Corp Armonk, NY, United States). The significance level was set to 0.05. All data showed normal distribution and homogeneous variance and were analyzed using two-way ANOVA, with independent levels and Bonferroni *post hoc* test.

## Results

### Bacteriophage Isolation and Propagation

**FIGURE 1:**
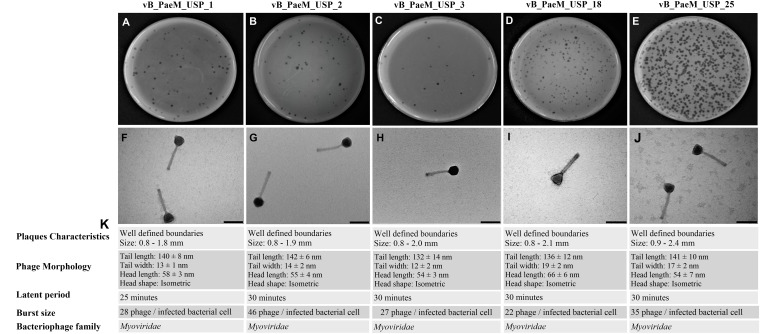
**(A–E)** Representative image of phage isolates depicting different plaque morphologies of vB_PaeM_USP_1, vB_PaeM_USP_2, vB_PaeM_USP_3, vB_PaeM_USP_18, and vB_PaeM_USP_25. **(F–J)** Transmission electron micrographs of individual bacteriophages. **(K)** Panel of plaque characteristics, phage morphology, latent period, and burst size.

Five new lytic bacteriophages against *P. aeruginosa* were isolated from domestic sewage and designated as vB_PaeM_USP_1, vB_PaeM_USP_2, vB_PaeM_USP_3, vB_PaeM_USP_18, and vB_PaeM_USP_25. All bacteriophages were able to form clear plaques, having well-defined boundaries. It was observed that none of the bacteriophages produced double haloes, and it was not possible to differentiate lysis plaques among the different isolated bacteriophages (Figures 1A–E).

### Electron Microscopy

The morphological aspects were slightly different among the different isolated viruses (Figures 1F–J). According to the International Committee on Taxonomy of Viruses, all bacteriophages belong to the order *Caudovirales* and are members of the *Myoviridae* family.

### Acid–Base and Thermal Stability

**FIGURE 2:**
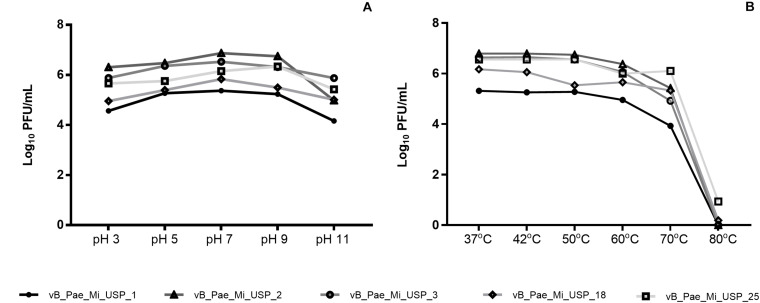
Effects of different pH and temperature values (log_10_^UFP/ml^) on vB_PaeM_USP_1, vB_PaeM_USP_2, vB_PaeM_USP_3, vB_PaeM_USP_18, and vB_PaeM_USP_25.

The values of pH 3.0 and pH 11.0 contributed to reduced bacteriophage viability (*p* < 0.05). Neutral pH (7.0) and small variations in hydrogenionic potential (pH 5.0 and pH 9.0) did not cause significant changes in viability. vB_PaeM_USP_2 and vB_PaeM_USP_3 were the most resistant to pH variation (Figure 2A). Reduced bacteriophage viability after exposure to 70 and 80°C was noticed. Bacteriophage vB_PaeM_USP_25 was the most resistant to temperature variation (Figure 2B).

### One-Step Growth Curve

A one-step growth curve revealed a similar behavior among the bacteriophages (Supplementary Figure 1). Latent period and yields at burst size are presented in Figure 1K.

### Genome Sequencing Analysis

**FIGURE 3:**
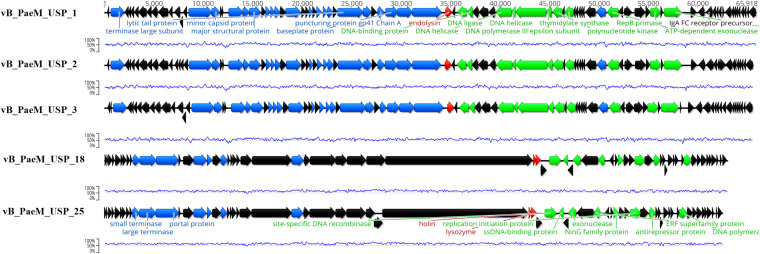
Genome map of the five sequenced bacteriophages depicting the predicted open reading frames colored according to their predicted function (black for hypothetical proteins, green for genes involved in DNA replication and transcription, blue for DNA packaging and morphogenesis genes, and red for cell lysis genes). The blue line represents the guanine-cytosine content (%).

**FIGURE 4:**
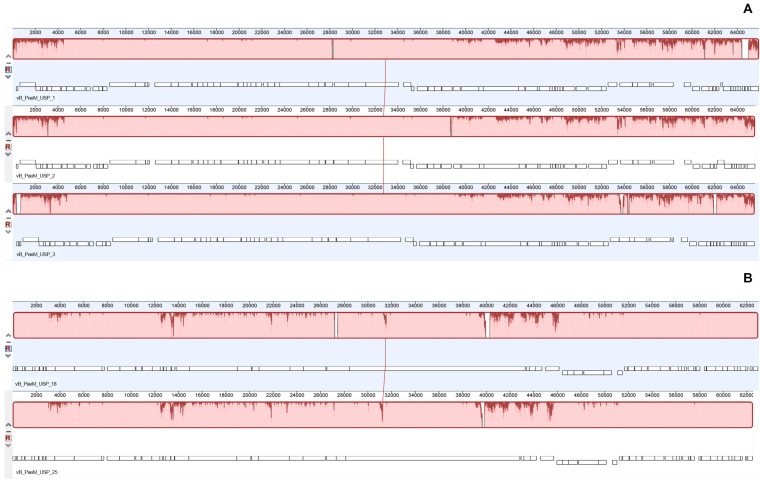
Whole genome alignment (using progressive Mauve algorithm) of the bacteriophage genomes demonstrating the high identity between the two groups. **(A)** Mauve alignment of the group containing bacteriophages vB_PaeM_USP_1, vB_PaeM_USP_2, and vB_PaeM_USP_3. **(B)** Mauve alignment of the group containing bacteriophages vB_PaeM_USP_18 and vB_PaeM_USP_25.

The complete genome sequence of each bacteriophage was deposited in the NCBI database under the accession numbers MT491204–MT491208. Genome analysis revealed that the five bacteriophages are potentially virulent since they do not encode for any known protein associated to lysogeny. Moreover, no known virulence-associated or toxic proteins were identified, revealing that the isolated bacteriophages are potentially safe for therapeutic purposes. All bacteriophage genomes are composed of double-stranded DNA molecules, and no putative genes coding for tRNAs were found. The general genomic properties of the five bacteriophages are presented in Table 2. Two different subgroups of bacteriophages could be observed (Figure 3). The first one, composed of bacteriophages vB_PaeM_USP_1, vB_PaeM_USP_2, and vB_PaeM_USP_3, presents similar genome sizes (approximately 65 kb), G + C content (approximately 55.5%), a high nucleotide pairwise identity (94.6%), and proteome homology (sharing 83 protein homologs between them), with roughly 62% of the proteins with unknown function (Figure 3). Despite their similarity, they present some differences at the beginning (0–5 kb) and at the end of their genome (53 kb end; Figure 4A). Their genomes share high nucleotide identity with other *P. aeruginosa* bacteriophage members of the *Pbunavirus* genus, namely, *Pseudomonas* virus Pa193, Epa22, vB_PaeM_PAO1_Ab27, and BrSP1, with identities above 96% and coverages of up to 98%.

**TABLE 2:** Genomic properties of *Pseudomonas aeruginosa* bacteriophages.

Phage	Accession number	Genome size (bp)	GC%	CDSs	Unknown proteins	Genus
vB_PaeM_USP_1	MT491204	65,918	55.6	87	54	*Pbunavirus*
vB_PaeM_USP_2	MT491205	65,606	55.5	86	53	*Pbunavirus*
vB_PaeM_USP_3	MT491206	65,567	55.5	89	56	*Pbunavirus*
vB_PaeM_USP_18	MT491207	63,030	63.7	65	48	Unknown
vB_PaeM_USP_25	MT491208	62,537	63.6	66	49	Unknown

The second subgroup, composed of vB_PaeM_USP_18 and vB_PaeM_USP_25, has nucleotide pairwise identity of 97.1% and shares 65 homologs, ca. 74% of them without an assigned function (Figure 3). A slightly lower homology is observed in the genomic regions 3–4.5, 12.5–14, and mainly from 39–46.5 kb (Figure 4B). The low homology with genomes from other phages deposited with the NCBI does not allow grouping these phage genomes with any known genus or subfamily.

### Host Range Investigation and EOP Analysis

All five bacteriophages exhibited a broad lytic spectrum and were able to infect 69.7% (*n* = 23) of the *P. aeruginosa* strains, of which seven were multidrug resistant. Furthermore, the bacteriophages showed lytic activity on clinical strains isolated from different infection sites (Table 1).

The EOP values of vB_PaeM_USP_1 (high: 13; insufficient: 6; low: 4), vB_PaeM_USP_2 (high: 14; insufficient: 6; low: 3), and vB_PaeM_USP_3 (high: 13; insufficient: 7; low: 3) were higher than vB_PaeM_USP_18 (high: 8; insufficient: 11; low: 4) and vB_PaeM_USP_25 (high: 6; insufficient: 11; low: 6). The last two viruses had low or inefficient EOP for all multidrug-resistant strains (Table 1). Although during host range investigation the bacteriophages had lysed the same strains, inefficient EOP results indicate that the haloes observed previously refer to “lysis from without events.”

### Infection of Planktonic Cells

**FIGURE 5:**
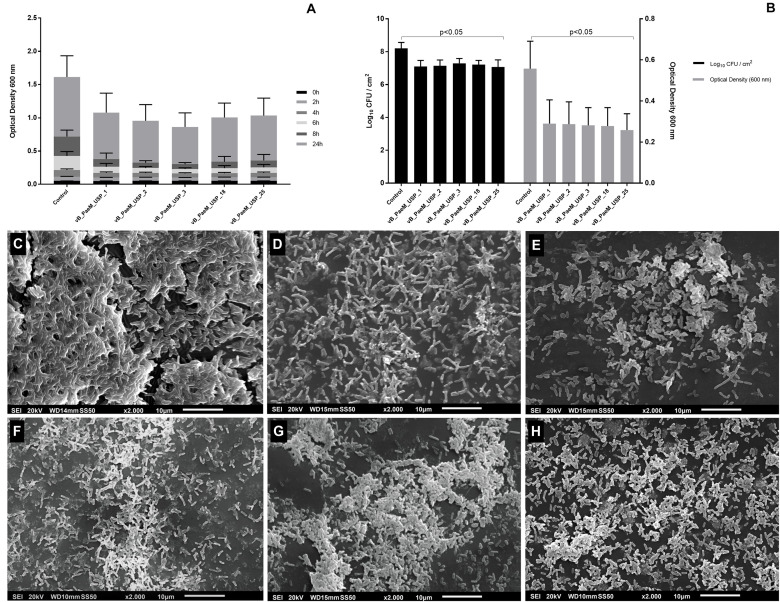
**(A)** Optical density of planktonic *Pseudomonas aeruginosa* strains after 2, 4, 6, 8, and 24 h of co-incubation with different bacteriophages. **(B)** Anti-biofilm activity of different bacteriophages on biofilm viability rates expressed in log_10_ CFU/cm^2^ (left *y* axis) and metabolic activity expressed in absorbance at 492 nm (right *y* axis). Inhibition of planktonic growth and anti-biofilm activity assays was carried out at three independent times in triplicate against 15 strains of *P. aeruginosa*. **(C–H)** Scanning electron micrographs after phage treatment: **(C)** control, **(D)** vB_PaeM_USP_1, **(E)** vB_PaeM_USP_2, **(F)** vB_PaeM_USP_3, **(G)** vB_PaeM_USP_18, and **(H)** vB_PaeM_USP_25. Scale bar = 100 nm. After the treatment of bacteriophages, it is possible to observe lower planktonic growth, biofilm viability rates, and metabolic activity. Disrupted biofilms and cell debris are also noticed in SEM images.

All five bacteriophages reduced bacterial growth up to 8 h of incubation in 13 strains (*p* < 0.05; Figure 5A). The reduction was above 60% after 6 and 8 h of incubation. After 24 h of incubation, all bacteriophages were able to maintain lower OD values in three strains, but with reductions in bacterial growth of 45% (*p* < 0.05). In five other strains, the growth rates were reduced by different bacteriophages, and the growth of five strains was not affected by the bacteriophages. Concerning multidrug-resistant *P. aeruginosa*, the bacteriophages promoted alteration in growth rates in one of the two strains. vB_PaeM_USP_1 and vB_PaeM_USP_3 promoted the greatest growth reductions (Supplementary Tables 1–5).

### Anti-biofilm Activity Measurement

All five bacteriophages reduced the biofilm viability rates in 12 strains (*p* < 0.05) in about 10-fold CFU/cm^2^ (Figure 5B). vB_PaeM_USP_2 and vB_PaeM_USP_18 presented increased anti-biofilm activity against two multidrug-resistant strains.

Concerning the metabolic activity (XTT), after treatment of the bacteriophages, the absorbance values reduced in about 50% in 11 strains (*p* < 0.05; Figure 5B). Bacteriophage vB_PaeM_USP_2 was able to significantly reduce the metabolic activity. The mean and the standard deviation values (CFU/cm^2^ and XTT) after exposure to different bacteriophages are shown in the Supplementary Data (Supplementary Tables 6, 7).

Results from SEM micrographs showed densely associated bacteria in the control biofilm (Figure 5C). In contrast, after exposure to bacteriophages, it is possible to observe disrupted biofilms and cell debris, demonstrating remarkable biofilm reduction (Figures 5D–H).

## Discussion

This study comprises the isolation and the full characterization of five new *P. aeruginosa* bacteriophages isolated from domestic sewage. The bacteriophages showed a broad lytic spectrum, able to disrupt *P. aeruginosa* biofilms on endotracheal tube surfaces. Based on the results, the null hypothesis was rejected since there was a statistical difference on planktonic bacteria growth, viability rates, and metabolic activity of the biofilm.

The vast majority of the already characterized bacteriophages are tailed (Hatfull and Hendrix, 2011). Through morphological and genomics analysis, here there is evidence that all the five bacteriophages have similar features, belonging to the order Caudovirales and *Myoviridae* family. Moreover, genome sequencing revealed that vB_PaeM_USP_1, vB_PaeM_USP_2, and vB_PaeM_USP_3 were most closely related to Epa22, sharing about 96% of identity. Their genomes are typically organized in clusters. Both guanine-cytosine content and genome length were similar to other Pbunaviruses as previously reported (de Melo et al., 2019; Yuan et al., 2019). Regarding vB_PaeM_USP_18 and vB_PaeM_USP_25, only ca. 20% of their genomes could be aligned with other phage genomes deposited at the NCBI. This low identity with other known genomes does not allow grouping these two phages with any known genus or subfamily. The inability to group these two genomes suggests that they belong to a new bacteriophage genus. Genomic bioinformatics analysis allowed identifying the endolysin gene on all bacteriophages. Commonly, this enzyme is responsible for the lytic ability of tailed bacteriophages (Wu et al., 2019). In addition, several open reading frames could only be annotated as coding for hypothetical proteins and thus with an unknown function, something that is common in bacteriophage studies (Guo et al., 2019).

The one-step growth curve analysis revealed that the latent period was similar in the five bacteriophages (about 30 min; Figure 1H), with a progeny per infected cell varying from 22 to 46 bacteriophage particles. Regarding the latent period, the same result was reported previously. Nonetheless, comparing burst sizes with bacteriophages with a similar genome, we noticed that all bacteriophages produced lower burst sizes (Guo et al., 2019; Yuan et al., 2019). The reduction observed here could be explained by differences in bacterial growth rates since the burst size increases linearly with bacterial growth rate (Nabergoj et al., 2018). Adsorption and latent period also vary with bacterial growth rate, decreasing at higher growth rates.

The infectivity of the bacteriophages remained stable for a pH range from 5.0 to 9.0 and temperature range from 37 to 60°C (Figure 2). Although pH and temperature do not undergo abrupt variation in physiological systems, comprehending this aspect is important in order to maintain bacteriophage viability and infectivity. Even though new antimicrobial agents should be investigated, the large pH and temperature tolerance indicate a broad workability range to search and apply bacteriophage-based products to setups of different infection models. For instance, skin becomes more alkaline by bacterial colonization, and wounds show pH variations in relation to healthy skin (Schneider et al., 2007).

The lytic activity of bacteriophages was tested against 37 strains (33 *P. aeruginosa* and four different bacterial species). Our results reveal that all bacteriophages could efficiently lyse 69.7% of the *P. aeruginosa* strains, of which seven were multidrug resistant. Infection of multidrug-resistant *P. aeruginosa* indicates that bacteriophages or bacteriophage-based products could potentially be applied for therapeutic purposes. The lytic rates presented here are higher than in other *Pbunavirus* (de Melo et al., 2019), which can be related to the origin and the number of tested strains. In fact, host broad range and species specificity are desirable for bacteriophage application in phage therapy. Moreover, bacteriophages should be strictly lytic in order to avoid the risk of potential transmission of virulent bacterial genes (Canchaya et al., 2003). Here five lytic bacteriophages had promising results in disrupting mature *P. aeruginosa* biofilms and can be further explored in studies involving phage therapy.

Efficiency of plating assays indicated a considerable variation from low to high between the different bacteriophages on the different strains. Moreover, the bacteriophages showed inefficient EOP for six strains. Differences in EOP and absence of lysis can be explained by the recognition process, which can diverge in different strains according to the expression of bacteriophage receptors on the bacterial outer membrane (Uchiyama et al., 2016). Furthermore, the localization, volume, and density of surface molecules can affect bacteriophage adsorption (Rakhuba et al., 2010). Considering the mentioned variation in molecules for bacteriophage adsorption, the application of a bacteriophage cocktail, which retains distinct adsorption mechanisms, is important to maintain bacteriophage infectivity (Forti et al., 2018). Apart from the recognition process, well-known bacterial resistance systems against bacteriophages could have led to low EOP values. Literature has pointed out that many antiviral mechanisms may be employed by the host cell to evade infection from bacteriophages, such as restriction nucleases and CRISPR (Seed, 2015).

Assessing the efficacy of bacteriophages in reducing *P. aeruginosa* growth in planktonic form, we observed significant reductions for up to 8 h of co-incubation for almost all investigated strains. However, following 24 h of co-incubation, only three strains showed reduced growth rates. Two multidrug-resistant *P. aeruginosa* did not show reduced growth rates at the evaluated times. Guo et al. (2019) demonstrated that two new phages at low MOI (0.01) had good performance both on preventing biofilm formation and eradicating preformed biofilms. Moreover, Melo et al. (2014), using a MOI of 0.05, demonstrated reduction in the optical density of planktonic cells in about 83% of clinical isolates after 24 h of infection. We considered that antiviral mechanisms might result in the emergence of bacteriophage-insensitive bacterial strains through blocking of bacteriophage receptors, production of extracellular matrix, production of competitive inhibitors, prevention of bacteriophage DNA entry, slicing of bacteriophage nucleic acids, and abortive infection mechanisms (Labrie et al., 2010).

We observed a great reduction in planktonic growth and anti-biofilm activity after treatment of bacteriophages, and it was widely coincident in both assays. Slight differences were detected among the strains, which can be related to distinct analysis methods. Planktonic assays were performed by OD, while anti-biofilm activity was evaluated by CFU counts and activity metabolic measurement. Comparing OD and CFU results, only one strain showed a discordant outcome. Considering the three analysis methods (OD, CFU, and XTT), the results of six strains did not seem to be concordant. The lytic activity of the bacteriophages was relevant to biofilm control on the endotracheal tube surface. Although no depolymerase was identified in these bacteriophage genomes, the anti-biofilm action could be related to the penetration of viruses at the deeper layers of the biofilm by means of the degradation of the polysaccharide matrix (Lin et al., 2017).

Anti-biofilm results support the hypothesis that bacteriophages might be applied as a biocontrol tool against multidrug-resistant *P. aeruginosa* on the endotracheal tube surface in the near future. Systemic treatment with antibiotics did not fully inhibit biofilm development on endotracheal tube surfaces (Fernández-Barat et al., 2012). Therefore, direct administration of non-antibiotic approaches, such as antimicrobial agents (Guillon et al., 2018) and bacteriophages, seem to be a suitable option for the fight against persistent biofilms. Indeed bacteriophages could be potentially applied on endotracheal tube biofilm as prophylactic and therapeutic agent. Bacteriophage-coated tubes could be attended to inhibit bacterial adhesion, whereas direct application may be useful in reducing microbial bioburden. Aleshkin et al. (2016) reported the intragastric administration of a phage cocktail in patients on mechanical ventilation, aiming for the treatment of acute infections caused by nosocomial pathogens. The authors demonstrated an important reduction in bacterial counts after phage therapy, indicating it as an alternative purpose to antibiotics to treat healthcare-associated infections.

Literature has presented that bacteriophages or the synergistic action of bacteriophage and antibiotics constitute promising strategies against multidrug-resistant *P. aeruginosa* biofilm (Akturk et al., 2019; Adnan et al., 2020). Given the ability of new lytic bacteriophages to disrupt and reduce the microbial load of endotracheal-associated biofilms *in vitro*, several aspects must be taken into consideration before the transfer of phage therapy to clinical practice. As the device is not in contact with vascularized tissues, potential bacteriophage administration routes should be discussed. Moreover, in clinical settings, it is highly unlikely that the endotracheal tubes will continuously be exposed to the phages, as demonstrated in this *in vitro* study. Thus, we suggest that phage therapy could be applied during endotracheal suctioning, as recommended for ventilated patients. The instillation of bacteriophages during the aspiration of pulmonary secretions could favor its direct application (Guillon et al., 2018). Inhalation of aerosol-containing antibiotics and phage formulations is another potential administration route.

In addition, it should be considered that *in vivo* biofilms are different from the *in vitro* ones studied here, probably due to the presence of body fluids, host cells, and different interactions among microorganisms. In light of the aforementioned assumptions, future investigations considering mixed biofilm models and the synergistic action of phage and antibiotic therapy should be conducted. Although treatment of bacteriophage reduced the concentration of *P. aeruginosa* both on planktonic and biofilm forms, further research is required to determine the correct concentration and the possible combinations of different bacteriophages to establish a cocktail. Based on EOP and anti-biofilm activity and considering the similarities among the five bacteriophages, we suggest that, in further studies, a cocktail composed of vB_PaeM_USP_2 and vB_PaeM_USP_18 should be used. Moreover, only one administration with a single bacteriophage concentration to treat biofilms was evaluated. Assessing different times of infection, a strong lytic activity could be evidenced. In addition, special attention should be paid to the potential development of bacteriophage resistance. In view of this, we also consider it necessary to evaluate the phenotype of bacteriophage-insensitive mutants after a long exposure to bacteriophages.

## Conclusion

Biofilms associated with endotracheal tubes are critical concerns in the development of VAP. Since antibiotic therapy is not profitable to eliminate biofilms on the surface of medical devices, bacteriophage therapy offers a possible alternative to reduce bacterial colonization on the surface of endotracheal tubes. The studied bacteriophages were efficient in controlling the growth of *P. aeruginosa* and promoted biofilm disruption on the surface of endotracheal tubes, including multidrug-resistant strains. Thus, they might be considered as good candidates for therapeutic studies and the application of bacteriophage-encoded proteins.

## Data Availability Statement

The datasets presented in this study can be found in online repositories. The names of the repository/repositories and accession number(s) can be found in the article/ Supplementary Material.

## Author Contributions

EW contributed to conceptualization and funding acquisition. VO carried out the main body of research and wrote the original manuscript. FB and RM contributed to the phage growth experiments. AM contributed to data analysis. CS-L and HP supervised the work’s progress and edited the manuscript. LM and SS contributed to bioinformatics analysis and edited the manuscript. All authors have read and agreed to the published version of the manuscript.

## Conflict of Interest

The authors declare that the research was conducted in the absence of any commercial or financial relationships that could be construed as a potential conflict of interest.
