# Supplementary figures and images for: Identification and Characterization of New Bacteriophages to Control Multidrug-Resistant Pseudomonas aeruginosa Biofilm on Endotracheal Tubes
Source: Front Microbiol. 2020 Oct 6;11:580779. doi: 10.3389/fmicb.2020.580779 (PMC7573221; doi:10.3389/fmicb.2020.580779)

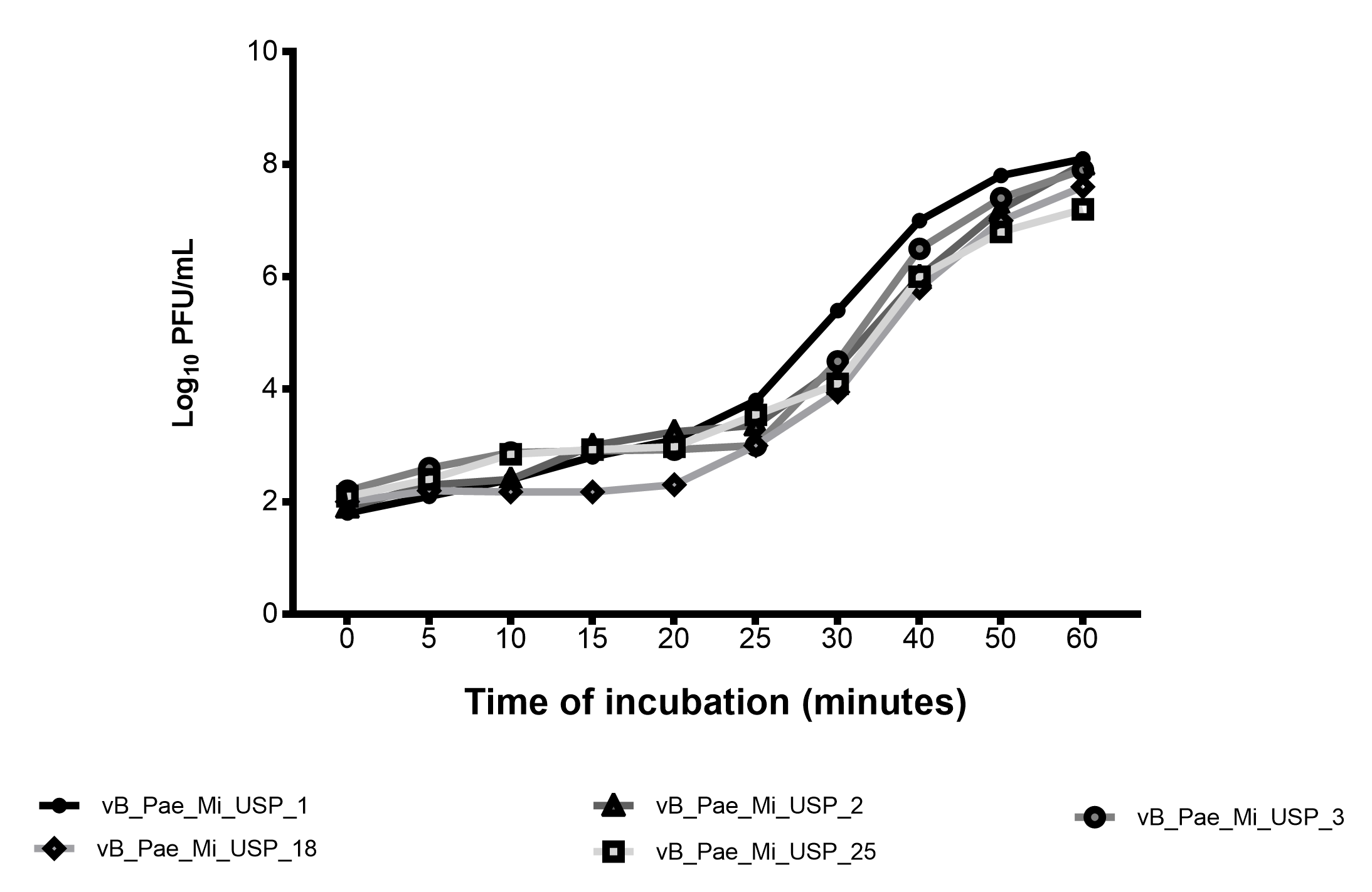

Supplement: Supplementary file 1 [file Image_1.TIF]
