# Supplementary material for: Identification and Characterization of New Bacteriophages to Control Multidrug-Resistant Pseudomonas aeruginosa Biofilm on Endotracheal Tubes
Source: Front Microbiol. 2020 Oct 6;11:580779. doi: 10.3389/fmicb.2020.580779 (PMC7573221; doi:10.3389/fmicb.2020.580779)
Supplement: Supplementary file 2 [file Data_Sheet_1.docx]

**Table S1:** Mean and standard deviation values of planktonic growth (OD_600_) of *P. aeruginosa* strains after 2 h of incubation with different bacteriophages.

| Clinical isolates and ATCC strains | **Control** | **vB_PaeM_USP_1** | **vB_PaeM_USP_2** | **vB_PaeM_USP_3** | **vB_PaeM_USP_18** | **vB_PaeM_USP_25** | Total  (Strains- 2 h) |
| --- | --- | --- | --- | --- | --- | --- | --- |
| Pa_Mi_1 | 0.061 (0.002) ^Bcd^ | 0.053 (0.001) ^Aa^ | 0.053 (0.001) ^Aab^ | 0.052 (0.001) ^Aa^ | 0.052 (0.001) ^Aa^ | 0.053 (0.002) ^Aa^ | 0.054 (0.004) |
| Pa_Mi_2 | 0.065 (0.003) ^Bde^ | 0.056 (0.006) ^Aa^ | 0.054 (0.003) ^Aabc^ | 0.052 (0.002) ^Aa^ | 0.053 (0.001) ^Aa^ | 0.054 (0.002) ^Aa^ | 0.056 (0.005) |
| Pa_Mi_6 | 0.063 (0.001) ^Cde^ | 0.055 (0.006) ^ABa^ | 0.054 (0.002) ^ABabc^ | 0.051 (0.001) ^Aa^ | 0.055 (0.005) ^ABab^ | 0.059 (0.011) ^BCbcd^ | 0.056 (0.007) |
| Pa_Mi_7 | 0.066 (0.002) ^Bde^ | 0.052 (0.003) ^Aa^ | 0.053 (0.002) ^Aab^ | 0.052 (0.002) ^Aa^ | 0.051 (0.001) ^Aa^ | 0.052 (0.003) ^Aa^ | 0.054 (0.006) |
| Pa_Ba_164 | 0.070 (0.004) ^Cef^ | 0.053 (0.002) ^Aa^ | 0.058 (0.008) ^ABabc^ | 0.052 (0.002) ^Aa^ | 0.057 (0.007) ^ABabc^ | 0.059 (0.009) ^Bcd^ | 0.058 (0.008) |
| Pa_Ba_168 | 0.070 (0.007) ^Cef^ | 0.052 (0.002) ^Aa^ | 0.060 (0.011) ^Bbc^ | 0.052 (0.001) ^Aa^ | 0.061 (0.014) ^Bbc^ | 0.056 (0.008) ^ABabcd^ | 0.058 (0.010) |
| Pa_Ba_169 | 0.060 (0.005) ^Bbcd^ | 0.052 (0.002) ^Aa^ | 0.053 (0.002) ^Aab^ | 0.051 (0.003) ^Aa^ | 0.054 (0.003) ^ABab^ | 0.057 (0.008) ^ABabcd^ | 0.054 (0.005) |
| Pa_Trac_20 | 0.074 (0.004) ^Def^ | 0.056 (0.007) ^ABa^ | 0.061 (0.008) ^BCc^ | 0.055 (0.004) ^Aa^ | 0.062 (0.008) ^Cc^ | 0.063 (0.011) ^Cc^ | 0.062 (0.009) |
| Pa_Trac_23 | 0.069 (0.005) ^Bef^ | 0.053 (0.002) ^Aa^ | 0.052 (0.002) ^Aa^ | 0.052 (0.002) ^Aa^ | 0.052 (0.001) ^Aa^ | 0.053 (0.002) ^Aabc^ | 0.055 (0.007) |
| Pa_Ren_1 | 0.060 (0.003) ^Bcd^ | 0.053 (0.005) ^Aa^ | 0.052 (0.003) ^Aa^ | 0.051 (0.001) ^Aa^ | 0.051 (0.001) ^Aa^ | 0.052 (0.002) ^Aabc^ | 0.053 (0.004) |
| Pa_ATCC 27853 | 0.065 (0.009) ^Bde^ | 0.049 (0.001) ^Aa^ | 0.051 (0.001) ^Aa^ | 0.049 (0.001) ^Aa^ | 0.051 (0.002) ^Aa^ | 0.051 (0.001) ^Aab^ | 0.053 (0.007) |
| Pa_ATCC 2108 | 0.054 (0.001) ^Aabc^ | 0.053 (0.001)^Aa^ | 0.053 (0.001) ^Aab^ | 0.053 (0.002) ^Aa^ | 0.053 (0.001) ^Aa^ | 0.053 (0.002) ^Aabc^ | 0.053 (0.002) |
| Pa_ATCC 2110 | 0.052 (0.001) ^Aa^ | 0.052 (0.001) ^Aa^ | 0.051 (0.001) ^Aa^ | 0.051 (0.001) ^Aa^ | 0.052 (0.001) ^Aa^ | 0.051 (0.001) ^Aa^ | 0.052 (0.001) |
| Pa_ATCC 2112 | 0.052 (0.001) ^Aab^ | 0.052 (0.001) ^Aa^ | 0.051 (0.001) ^Aa^ | 0.051 (0.001) ^Aa^ | 0.052 (0.001) ^Aa^ | 0.051 (0.001) ^Aab^ | 0.052 (0.001) |
| Pa_ATCC 2113 | 0.052 (0.001) ^Aa^ | 0.054 (0.009) ^Aa^ | 0.051 (0.002) ^Aa^ | 0.052 (0.004) ^Aa^ | 0.051 (0.001) ^Aa^ | 0.052 (0.001) ^Aabc^ | 0.052 (0.004) |
| Total (Phages) | 0.062 (0.008) | 0.053 (0.004) | 0.054 (0.005) | 0.052 (0.002) | 0.054 (0.006) | 0.054 (0.006) |  |

Comparisons of planktonic growth at 2 h were conducted be ANOVA two way with independent levels (strains and bacteriophage) and Bonferroni post-hoc test. ^ABCDE^ Different capital letters indicated statistically significant difference among bacteriophages. ^abcde^ Different small letters indicated statistically significant difference among strains.

**Table S2:** Mean and standard deviation values of planktonic growth (OD_600_) of *P. aeruginosa* strains after 4 h of incubation with different bacteriophages.

| Clinical isolates and ATCC strains | **Control** | **vB_PaeM_USP_1** | **vB_PaeM_USP_2** | **vB_PaeM_USP_3** | **vB_PaeM_USP_18** | **vB_PaeM_USP_25** | Total  (Strains- 4 h) |
| --- | --- | --- | --- | --- | --- | --- | --- |
| Pa_Mi_1 | 0.094 (0.006) ^Bcde^ | 0.052 (0.002) ^Aa^ | 0.052 (0.002) ^Aa^ | 0.051 (0.002) ^Aab^ | 0.052 (0.002) ^Aab^ | 0.051 (0.001) ^Aa^ | 0.059 (0.016) |
| Pa_Mi_2 | 0.107 (0.011) ^Bef^ | 0.065 (0.006) ^Aabcd^ | 0.058 (0.002) ^Aab^ | 0.056 (0.001) ^Aabc^ | 0.051 (0.002) ^Aab^ | 0.053 (0.002) ^Aab^ | 0.065 (0.020) |
| Pa_Mi_6 | 0.122 (0.008) ^Cfg^ | 0.065 (0.013) ^Aabcd^ | 0.058 (0.005) ^Aab^ | 0.067 (0.013) ^Abcd^ | 0.071 (0.012) ^ABcdef^ | 0.084 (0.022) ^Bde^ | 0.078 (0.025) |
| Pa_Mi_7 | 0.110 (0.009) ^Befg^ | 0.051 (0.001) ^Aa^ | 0.052 (0.003) ^Aa^ | 0.053 (0.002) ^Aab^ | 0.051 (0.003) ^Aab^ | 0.052 (0.003) ^Aa^ | 0.062 (0.022) |
| Pa_Ba_164 | 0.126 (0.020) ^Dg^ | 0.094 (0.021) ^Cfg^ | 0.084 (0.013) ^BCd^ | 0.071 (0.007) ^ABcd^ | 0.062 (0.005) ^Aabc^ | 0.070 (0.008) ^ABbcd^ | 0.085 (0.025) |
| Pa_Ba_168 | 0.121 (0.022) ^Cfg^ | 0.096 (0.018) ^Bg^ | 0.080 (0.015) ^Acd^ | 0.073 (0.008) ^Acd^ | 0.066 (0.006) ^Aabcd^ | 0.076 (0.011) ^Acde^ | 0.085 (0.023) |
| Pa_Ba_169 | 0.106 (0.025) ^Def^ | 0.087 (0.012) ^Cefg^ | 0.076 (0.013) ^BCcd^ | 0.066 (0.006) ^ABbcd^ | 0.061 (0.005) ^Aabc^ | 0.067 (0.009) ^ABabcd^ | 0.077 (0.020) |
| Pa_Trac_20 | 0.107 (0.008) ^Def^ | 0.071 (0.019) ^Abcde^ | 0.066 (0.019) ^Aabc^ | 0.072 (0.020) ^ABcd^ | 0.086 (0.024) ^BCf^ | 0.090 (0.029)^Ce^ | 0.082 (0.024) |
| Pa_Trac_23 | 0.116 (0.005) ^Bfg^ | 0.059 (0.010) ^Aabc^ | 0.059 (0.010) ^Aab^ | 0.060 (0.010) ^Aabcd^ | 0.059 (0.010) ^Aabc^ | 0.060 (0.010) ^Aabc^ | 0.069 (0.023) |
| Pa_Ren_1 | 0.084 (0.009) ^Bbcd^ | 0.053 (0.003) ^Aa^ | 0.050 (0.001) ^Aa^ | 0.052 (0.001) ^Aab^ | 0.050 (0.001) ^Aab^ | 0.052 (0.001)^Aa^ | 0.057 (0.013) |
| Pa_ATCC 27853 | 0.098 (0.015) ^Bde^ | 0.051 (0.001) ^Aa^ | 0.050 (0.001) ^Aa^ | 0.051 (0.001) ^Aab^ | 0.050 (0.002) ^Aa^ | 0.050 (0.001)^Aa^ | 0.058 (0.019) |
| Pa_ATCC 2108 | 0.075 (0.005) ^Aab^ | 0.077 (0.002) ^Adef^ | 0.075 (0.003) ^Abcd^ | 0.076 (0.003) ^Ad^ | 0.076 (0.004) ^Acdef^ | 0.076 (0.003) ^Acde^ | 0.076 (0.003) |
| Pa_ATCC 2110 | 0.065 (0.004) ^Aa^ | 0.055 (0.001) ^Aab^ | 0.056 (0.003) ^Aa^ | 0.052 (0.001) ^Aab^ | 0.053 (0.001) ^Aab^ | 0.053 (0.001)^Aab^ | 0.056 (0.005) |
| Pa_ATCC 2112 | 0.109 (0.015) ^Cefg^ | 0.076 (0.004) ^Bcde^ | 0.082 (0.004) ^Bcd^ | 0.048 (0.001)^A a^ | 0.081 (0.006) ^Bdef^ | 0.078 (0.005) ^Bde^ | 0.079 (0.019) |
| Pa_ATCC 2113 | 0.081 (0.010) ^Aabc^ | 0.073 (0.014) ^Acde^ | 0.080 (0.013) ^Acd^ | 0.073 (0.012) ^Acd^ | 0.067 (0.012) ^Abcde^ | 0.076 (0.013) ^Acde^ | 0.075 (0.013) |
| Total (Phages) | 0.101 (0.022) | 0.068 (0.018) | 0.065 (0.015) | 0.061 (0.012) | 0.062 (0.014) | 0.066 (0.017) |  |

Comparisons of planktonic growth at 4 h were conducted be ANOVA two way with independent levels (strains and bacteriophage) and Bonferroni post-hoc test. ^ABCDE^ Different capital letters indicated statistically significant difference among bacteriophages. ^abcde^ Different small letters indicated statistically significant difference among strains.

**Table S3:** Mean and standard deviation values of planktonic growth (OD_600_) of *P. aeruginosa* strains after 6 h of incubation with different bacteriophages.

| Clinical isolates and ATCC strains | **Control** | **vB_PaeM_USP_1** | **vB_PaeM_USP_2** | **vB_PaeM_USP_3** | **vB_PaeM_USP_18** | **vB_PaeM_USP_25** | Total  (Strains- 6 h) |
| --- | --- | --- | --- | --- | --- | --- | --- |
| Pa_Mi_1 | 0.208 (0.058) ^Bdef^ | 0.053 (0.002) ^Aab^ | 0.051 (0.002) ^Aa^ | 0.052 (0.002) ^Aab^ | 0.051 (0.002) ^Aa^ | 0.052 (0.002) ^Aa^ | 0.078 (0.063) |
| Pa_Mi_2 | 0.225 (0.028) ^Bdefg^ | 0.073 (0.019) ^Aabc^ | 0.057 (0.002) ^Aab^ | 0.053 (0.001) ^Aab^ | 0.051 (0.002) ^Aa^ | 0.053 (0.002) ^Aa^ | 0.085 (0.065) |
| Pa_Mi_6 | 0.323 (0.036) ^Dj^ | 0.067 (0.016) ^Aabc^ | 0.056 (0.005) ^Aab^ | 0.089 (0.019) ^ABbcd^ | 0.116 (0.009) ^Bc^ | 0.169 (0.043) ^Cde^ | 0.136 (0.095) |
| Pa_Mi_7 | 0.251 (0.084) ^Bgh^ | 0.056 (0.007) ^Aabc^ | 0.056 (0.008) ^Aab^ | 0.057 (0.008) ^Aabc^ | 0.056 (0.009) ^Aa^ | 0.059 (0.013) ^Aa^ | 0.089 (0.081) |
| Pa_Ba_164 | 0.296 (0.021) ^Dij^ | 0.195 (0.066) ^Be^ | 0.106 (0.024) ^Cef^ | 0.067 (0.004) ^Aabcd^ | 0.055 (0.003) ^Aa^ | 0.064 (0.007) ^Aa^ | 0.130 (0.093) |
| Pa_Ba_168 | 0.267 (0.016) ^Dhi^ | 0.188 (0.060) ^Be^ | 0.118 (0.031) ^Cf^ | 0.078 (0.008) ^Aabcd^ | 0.062 (0.005) ^Aa^ | 0.076 (0.013) ^Aab^ | 0.131 (0.079) |
| Pa_Ba_169 | 0.245 (0.028) ^Dfgh^ | 0.167 (0.048) ^Ce^ | 0.098 (0.018) ^Bcdef^ | 0.065 (0.006) ^ABabcd^ | 0.057 (0.003) ^Aa^ | 0.063 (0.007) ^Aa^ | 0.116 (0.073) |
| Pa_Trac_20 | 0.159 (0.017) ^Cb^ | 0.093 (0.048) ^Acd^ | 0.092 (0.053) ^Abcdef^ | 0.096 (0.057) ^Acd^ | 0.124 (0.043) ^ABc^ | 0.132 (0.054) ^Bcd^ | 0.116 (0.051) |
| Pa_Trac_23 | 0.227 (0.046) ^Befgh^ | 0.064 (0.010) ^Aabc^ | 0.061 (0.008) ^Aabc^ | 0.065 (0.010) ^Aabcd^ | 0.063 (0.009) ^Aa^ | 0.062 (0.009) ^Aa^ | 0.090 (0.065) |
| Pa_Ren_1 | 0.204 (0.044) ^Bcde^ | 0.054 (0.003) ^Aabc^ | 0.051 (0.002) ^Aa^ | 0.052 (0.001) ^Aab^ | 0.050 (0.001) ^Aa^ | 0.051 (0.002) ^Aa^ | 0.077 (0.060) |
| Pa_ATCC 27853 | 0.186 (0.030) ^Bcd^ | 0.049 (0.001) ^Aa^ | 0.049 (0.001) ^Aa^ | 0.049 (0.001) ^Aab^ | 0.049 (0.002) ^Aa^ | 0.049 (0.001) ^Aa^ | 0.072 (0.053) |
| Pa_ATCC 2108 | 0.104 (0.008) ^Aa^ | 0.113 (0.003) ^Ad^ | 0.104 (0.006) ^Adef^ | 0.103 (0.005) ^Ad^ | 0.103 (0.006) ^Abc^ | 0.105 (0.004) ^Abc^ | 0.105 (0.006) |
| Pa_ATCC 2110 | 0.085 (0.007) ^Aa^ | 0.058 (0.001) ^Aabc^ | 0.065 (0.014) ^Aabcd^ | 0.055 (0.001) ^Aab^ | 0.056 (0.001) ^Aa^ | 0.056 (0.002) ^Aa^ | 0.063 (0.012) |
| Pa_ATCC 2112 | 0.258 (0.047) ^Cghi^ | 0.091 (0.007) ^Bbcd^ | 0.092 (0.020) ^Bbcdef^ | 0.048 (0.001) ^Aa^ | 0.071 (0.007) ^ABab^ | 0.070 (0.007) ^ABab^ | 0.105 (0.073) |
| Pa_ATCC 2113 | 0.165 (0.012) ^Bbc^ | 0.066 (0.008) ^Aabc^ | 0.066 (0.005) ^Aabcde^ | 0.078 (0.004) ^Aabcd^ | 0.182 (0.010) ^Bd^ | 0.189 (0.014) ^Bd^ | 0.124 (0.056) |
| Total  Total (Phages) | 0.213 (0.073) | 0.092 (0.056) | 0.075 (0.029) | 0.067 (0.023) | 0.076 (0.039) | 0.083 (0.047) |  |

Comparisons of planktonic growth at 6 h were conducted be ANOVA two way with independent levels (strains and bacteriophage) and Bonferroni post-hoc test. ^ABCDE^ Different capital letters indicated statistically significant difference among bacteriophages. ^abcde^ Different small letters indicated statistically significant difference among strains.

**Table S4:** Mean and standard deviation values of planktonic growth (OD_600_) of *P. aeruginosa* strains after 8 h of incubation with different bacteriophages.

| Clinical isolates and ATCC strains | **Control** | **vB_PaeM_USP_1** | **vB_PaeM_USP_2** | **vB_PaeM_USP_3** | **vB_PaeM_USP_18** | **vB_PaeM_USP_25** | Total  (Strains- 8 h) |
| --- | --- | --- | --- | --- | --- | --- | --- |
| Pa_Mi_1 | 0.317 (0.034) ^Bcd^ | 0.052 (0.002) ^Aa^ | 0.051 (0.002) ^Aab^ | 0.052 (0.002) ^Aa^ | 0.052 (0.002) ^Aa^ | 0.052 (0.002) ^Aa^ | 0.096 (0.101) |
| Pa_Mi_2 | 0.307 (0.054) ^Bcd^ | 0.083 (0.027) ^Aab^ | 0.058 (0.002) ^Aabc^ | 0.056 (0.003) ^Aab^ | 0.057 (0.004) ^Aa^ | 0.057 (0.003) ^Aa^ | 0.103 (0.096) |
| Pa_Mi_6 | 0.450 (0.115) ^Df^ | 0.070 (0.014) ^Aa^ | 0.058 (0.004) ^Aabc^ | 0.096 (0.023) ^Aabc^ | 0.152 (0.018) ^Bc^ | 0.253 (0.044) ^Cd^ | 0.180 (0.147) |
| Pa_Mi_7 | 0.327 (0.117) ^Bcd^ | 0.076 (0.039) ^Aab^ | 0.074 (0.035) ^Aabcd^ | 0.074 (0.032) ^Aabc^ | 0.069 (0.029) ^Aab^ | 0.076 (0.036) ^Aab^ | 0.116 (0.110) |
| Pa_Ba_164 | 0.349 (0.085) ^Cde^ | 0.305 (0.035) ^Cc^ | 0.111 (0.023) ^Babcd^ | 0.060 (0.003) ^ABabc^ | 0.053 (0.002) ^Aa^ | 0.061 (0.008) ^ABab^ | 0.156 (0.129) |
| Pa_Ba_168 | 0.351 (0.074) ^Dde^ | 0.284 (0.025) ^Cc^ | 0.135 (0.025) ^Bd^ | 0.072 (0.008) ^Aabc^ | 0.057 (0.004) ^Aa^ | 0.071 (0.016) ^Aab^ | 0.161 (0.120) |
| Pa_Ba_169 | 0.331 (0.090) ^Ccd^ | 0.255 (0.032) ^Bc^ | 0.103 (0.020) ^Aabcd^ | 0.059 (0.004) ^Aabc^ | 0.055 (0.003) ^Aa^ | 0.059 (0.007) ^Aab^ | 0.144 (0.117) |
| Pa_Trac_20 | 0.172 (0.018) ^Bab^ | 0.126 (0.058) ^ABb^ | 0.115 (0.055) ^Abcd^ | 0.120 (0.062) ^ABbc^ | 0.155 (0.035) ^ABc^ | 0.171 (0.055) ^ABc^ | 0.143 (0.053) |
| Pa_Trac_23 | 0.404 (0.100) ^Bef^ | 0.080 (0.022) ^Aab^ | 0.079 (0.022) ^Aacd^ | 0.087 (0.027) ^Aabc^ | 0.081 (0.024) ^Aab^ | 0.081 (0.024) ^Aab^ | 0.135 (0.129) |
| Pa_Ren_1 | 0.274 (0.058) ^Bc^ | 0.054 (0.004) ^Aa^ | 0.053 (0.003) ^Aab^ | 0.053 (0.003) ^Aa^ | 0.051 (0.001) ^Aa^ | 0.053 (0.003) ^Aa^ | 0.090 (0.086) |
| Pa_ATCC 27853 | 0.217 (0.090) ^Bb^ | 0.049 (0.001) ^Aa^ | 0.049 (0.001) ^Aa^ | 0.050 (0.001) ^Aa^ | 0.051 (0.002) ^Aa^ | 0.051 (0.002) ^Aa^ | 0.078 (0.072) |
| Pa_ATCC 2108 | 0.128 (0.008) ^Aa^ | 0.135 (0.005) ^Ab^ | 0.123 (0.004) ^Acd^ | 0.122 (0.005) ^Ac^ | 0.124 (0.006) ^Abc^ | 0.124 (0.004) ^Abc^ | 0.126 (0.007) |
| Pa_ATCC 2110 | 0.113 (0.015) ^Aa^ | 0.075 (0.002) ^Aab^ | 0.084 (0.040) ^Aabcd^ | 0.067 (0.004) ^Aabc^ | 0.068 (0.005) ^Aab^ | 0.068 (0.006) ^Aab^ | 0.079 (0.024) |
| Pa_ATCC 2112 | 0.292 (0.185) ^Bcd^ | 0.059 (0.003) ^Aa^ | 0.065 (0.006) ^Aabc^ | 0.049 (0.001) ^Aa^ | 0.058 (0.003) ^Aab^ | 0.057 (0.003) ^Aa^ | 0.097 (0.114) |
| Pa_ATCC 2113 | 0.341 (0.019) ^Bde^ | 0.055 (0.002) ^Aa^ | 0.060 (0.004) ^Aabc^ | 0.073 (0.007) ^Aabc^ | 0.356 (0.022) ^Bd^ | 0.354 (0.014) ^Be^ | 0.206 (0.146) |
| Total  Total (Phages) | 0.292 (0.123) | 0.117 (0.089) | 0.081 (0.036) | 0.073 (0.030) | 0.096 (0.079) | 0.106 (0.088) |  |

Comparisons of planktonic growth at 8 h were conducted be ANOVA two way with independent levels (strains and bacteriophage) and Bonferroni post-hoc test. ^ABCDE^ Different capital letters indicated statistically significant difference among bacteriophages. ^abcde^ Different small letters indicated statistically significant difference among strains.

**Table S5:** Mean and standard deviation values of planktonic growth (OD_600_) of *P. aeruginosa* strains after 24 h of incubation with different bacteriophages.

| Clinical and ATCC strains | **Control** | **vB_PaeM_USP_1** | **vB_PaeM_USP_2** | **vB_PaeM_USP_3** | **vB_PaeM_USP_18** | **vB_PaeM_USP_25** | Total  (Strains- 24 h) |
| --- | --- | --- | --- | --- | --- | --- | --- |
| Pa_Mi_1 | 1.015 (0.311) ^Befg^ | 0.462 (0.156) ^Ab^ | 0.447 (0.107) ^Ab^ | 0.424 (0.101) ^Aabc^ | 0.440 (0.085) ^Aab^ | 0.366 (0.125) ^Aa^ | 0.526 (0.273) |
| Pa_Mi_2 | 1.216 (0.076) ^Bghi^ | 0.499 (0.081) ^Abcd^ | 0.551 (0.099) ^Abc^ | 0.576 (0.141) ^Abcde^ | 0.608 (0.089) ^Aabcd^ | 0.585 (0.099) ^Aabcd^ | 0.672 (0.265) |
| Pa_Mi_6 | 1.298 (0.043) ^Di^ | 0.929 (0.143) ^Bef^ | 0.619 (0.154) ^Abcd^ | 0.648 (0.298) ^Acde^ | 0.999 (0.099) ^BCfg^ | 1.216 (0.083) ^Cf^ | 0.952 (0.300) |
| Pa_Mi_7 | 0.448 (0.133) ^Aab^ | 0.490 (0.181) ^Abc^ | 0.420 (0.177) ^Ab^ | 0.454 (0.149) ^Abcd^ | 0.471 (0.169) ^Aabc^ | 0.567 (0.225) ^Aabcd^ | 0.475 (0.173) |
| Pa_Ba_164 | 1.286 (0.066) ^Chi^ | 1.091 (0.049) ^Cf^ | 0.850 (0.166) ^Bde^ | 0.612 (0.155) ^Acde^ | 0.764 (0.165) ^ABdef^ | 0.802 (0.206) ^ABcde^ | 0.901 (0.265) |
| Pa_Ba_168 | 1.274 (0.071) ^Cghi^ | 1.119 (0.079) ^BCf^ | 0.981 (0.058) ^Be^ | 0.512 (0.076) ^Abcde^ | 0.624 (0.155) ^Aabcd^ | 0.515 (0.058) ^Aab^ | 0.837 (0.316) |
| Pa_Ba_169 | 1.185 (0.061) ^Dghi^ | 1.049 (0.084) ^Df^ | 0.853 (0.150) ^BCde^ | 0.556 (0.174) ^Abcde^ | 0.667 (0.146) ^ABbcd^ | 0.553 (0.153) ^Aabc^ | 0.810 (0.276) |
| Pa_Trac_20 | 0.680 (0.248) ^Abcd^ | 0.733 (0.159) ^Acde^ | 0.802 (0.067) ^Acde^ | 0.770 (0.098) ^Ae^ | 0.628 (0.299) ^Aabcd^ | 0.822 (0.076) ^Ade^ | 0.739 (0.185) |
| Pa_Trac_23 | 0.876 (0.502) ^Adef^ | 1.054 (0.132) ^ABf^ | 0.957 (0.306) ^ABe^ | 1.081 (0.236) ^ABf^ | 1.103 (0.143) ^Bg^ | 1.118 (0.059) ^Bf^ | 1.031 (0.273) |
| Pa_Ren_1 | 0.758 (0.485) ^Bcde^ | 0.476 (0.229) ^Abc^ | 0.573 (0.117) ^ABbc^ | 0.649 (0.212) ^ABcde^ | 0.703 (0.092) ^Bbcde^ | 0.586 (0.103) ^ABabcd^ | 0.624 (0.253) |
| Pa_ATCC 27853 | 0.508 (0.356) ^Aabc^ | 0.565 (0.120) ^Abcd^ | 0.485 (0.102) ^Ab^ | 0.409 (0.112) ^A abc^ | 0.451 (0.099) ^Aabc^ | 0.459 (0.137) ^Aab^ | 0.480 (0.178) |
| Pa_ATCC 2108 | 0.388 (0.069) ^Aa^ | 0.432 (0.030) ^Aab^ | 0.375 (0.042) ^Ab^ | 0.312 (0.039) ^Aab^ | 0.379 (0.024) ^Aa^ | 0.362 (0.023) ^Aa^ | 0.375 (0.053) |
| Pa_ATCC 2110 | 0.828 (0.080) ^Adef^ | 0.755 (0.051) ^Ade^ | 0.792 (0.124) ^Acde^ | 0.704 (0.063) ^Aef^ | 0.714 (0.026) ^Acde^ | 0.714 (0.039) ^Abcde^ | 0.751 (0.082) |
| Pa_ATCC 2112 | 0.630 (0.093) ^Aabcd^ | 0.656 (0.116) ^Abcd^ | 0.635 (0.120) ^Aacde^ | 0.518 (0.121) ^Abcde^ | 0.465 (0.178) ^Aabc^ | 0.473 (0.171) ^Aab^ | 0.563 (0.153) |
| Pa_ATCC 2113 | 1.034 (0.036) ^Bfgh^ | 0.172 (0.141) ^Aa^ | 0.104 (0.037) ^Aa^ | 0.161 (0.130) ^Aa^ | 0.960 (0.208) ^Befg^ | 0.971 (0.125) ^Bef^ | 0.567 (0.443) |
| Total  Total (Phages) | 0.895 (0.382) | 0.699 (0.308) | 0.630 (0.270) | 0.559 (0.253) | 0.665 (0.254) | 0.674 (0.281) |  |

Comparisons of planktonic growth at 24 h were conducted be ANOVA two way with independent levels (strains and bacteriophage) and Bonferroni post-hoc test. ^ABCDE^ Different capital letters indicated statistically significant difference among bacteriophages. ^abcde^ Different small letters indicated statistically significant difference among strains.

**Table S6:** Mean and standard deviation values of biofilm viabitily rates (Log_10_CFU/cm^2^) of *P. aeruginosa* mature biofilms after 24 h of incubation with different bacteriophages.

| Clinical isolates and ATCC strains | **Control** | **vB_PaeM_USP_1** | **vB_PaeM_USP_2** | **vB_PaeM_USP_3** | **vB_PaeM_USP_18** | **vB_PaeM_USP_25** | Total  (Strains – CFU/mL) |
| --- | --- | --- | --- | --- | --- | --- | --- |
| Pa_Mi_1 | 8.19 (0.49) ^Babcd^ | 7.13 (0.43) ^Aabcdef^ | 7.12 (0.32) ^Aabcde^ | 7.29 (0.33) ^Aabc^ | 7.23 (0.67) ^Aab^ | 6.85 (0.32) ^Aabc^ | 7.30 (0.60) |
| Pa_Mi_2 | 8.75 (0.53) ^Cd^ | 6.60 (0.42) ^ABa^ | 6.41 (0.63) ^Aa^ | 7.11 (0.48) ^Babc^ | 6.91 (0.31) ^ABab^ | 6.73 (0.55) ^ABab^ | 7.08 (0.91) |
| Pa_Mi_6 | 8.09 (0.53) ^Babcd^ | 6.97 (0.53) ^Aabcdef^ | 7.20 (0.32) ^Abcde^ | 7.30 (0.58) ^Aabc^ | 7.21 (0.58) ^Aab^ | 6.80 (0.21) ^Aab^ | 7.26 (0.61) |
| Pa_Mi_7 | 7.75 (0.65) ^Ba^ | 6.59 (0.39) ^Aa^ | 6.88 (0.35) ^Aabcd^ | 6.95 (0.29) ^Aab^ | 6.64 (0.37) ^Aa^ | 6.36 (0.35) ^Aa^ | 6.86 (0.60) |
| Pa_Ba_164 | 8.55 (0.21) ^Bbcd^ | 7.42 (0.42) ^Abcdef^ | 7.42 (0.24) ^Acde^ | 7.48 (0.52) ^Abc^ | 7.47 (0.34) ^Ab^ | 7.54 (0.63) ^Acde^ | 7.65 (0.57) |
| Pa_Ba_168 | 8.70 (0.45) ^Bcd^ | 7.59 (0.44) ^Adef^ | 7.65 (0.34) ^Ae^ | 7.59 (0.31) ^Abc^ | 7.57 (0.31) ^Ab^ | 7.85 (0.50) ^Ae^ | 7.82 (0.56) |
| Pa_Ba_169 | 8.54 (0.62) ^Bbcd^ | 7.67 (0.18) ^Af^ | 7.68 (0.18) ^Ae^ | 7.72 (0.37) ^Ac^ | 7.56 (0.48) ^Ab^ | 7.72 (0.27) ^Ade^ | 7.81 (0.50) |
| Pa_Trac_20 | 7.95 (0.26) ^Bab^ | 7.12 (0.27) ^Aabcdef^ | 7.26 (0.46) ^Abcde^ | 7.43 (0.48) ^ABbc^ | 7.17 (0.48) ^Aab^ | 7.03 (0.58) ^Aabcd^ | 7.33 (0.52) |
| Pa_Trac_23 | 8.01 (0.33) ^Babc^ | 6.70 (0.22) ^Aab^ | 6.74 (0.44) ^Aabc^ | 6.69 (0.33) ^Aa^ | 7.12 (0.27) ^Aab^ | 6.78 (0.35) ^Aab^ | 7.01 (0.57) |
| Pa_Ren_1 | 7.70 (0.39) ^Ca^ | 6.98 (0.53) ^ABabcdef^ | 7.13 (0.43) ^ABCabcde^ | 7.24 (0.40) ^ABCabc^ | 7.49 (0.53) ^BCb^ | 6.87 (0.27) ^Aabc^ | 7.23 (0.51) |
| Pa_ATCC 27853 | 8.63 (0.90) ^Cbcd^ | 7.46 (0.43) ^ABcdef^ | 7.48 (0.54) ^ABde^ | 7.79 (0.37) ^Bc^ | 7.40 (0.33) ^ABb^ | 7.02 (0.22) ^Aabcd^ | 7.63 (0.70) |
| Pa_ATCC 2108 | 7.72 (0.45) ^Ba^ | 7.62 (0.17) ^ABef^ | 7.36 (0.35) ^ABbcde^ | 7.32 (0.28) ^ABabc^ | 7.04 (0.22) ^Aab^ | 7.59 (0.43) ^ABde^ | 7.44 (0.39) |
| Pa_ATCC 2110 | 8.26 (0.68) ^Babcd^ | 6.92 (0.77) ^Aabcde^ | 7.13 (0.59) ^Aabcde^ | 6.96 (0.33) ^Aab^ | 7.18 (0.45) ^Aab^ | 6.84 (0.34) ^Aabc^ | 7.21 (0.72) |
| Pa_ATCC 2112 | 8.01 (0.58) ^Cabc^ | 6.81 (0.34) ^ABabc^ | 6.65 (0.35) ^Aab^ | 7.33 (0.45) ^Babc^ | 7.19 (0.42) ^ABab^ | 7.30 (0.44) ^Bbcde^ | 7.22 (0.61) |
| Pa_ATCC 2113 | 8.22 (0.34) ^Babcd^ | 6.89 (0.50) ^Aabcd^ | 6.91 (0.33) ^Aabcd^ | 7.15 (0.37) ^Aabc^ | 6.98 (0.42) ^Aab^ | 6.77 (0.39) ^Aab^ | 7.15 (0.62) |
| Total  Total (Phages) | 8.20 (0.61) | 7.10 (0.54) | 7.13 (0.52) | 7.29 (0.48) | 7.21 (0.48) | 7.07 (0.57) |  |

Comparisons of biofilm rates were conducted be ANOVA two way with independent levels (strains and bacteriophage) and Bonferroni post-hoc test. ^ABCDE^ Different capital letters indicated statistically significant difference among bacteriophages. ^abcde^ Different small letters indicated statistically significant difference among strains.

**Table S7:** Mean and standard deviation values of metabolic activity (absorbance - 492 nm) of *P. aeruginosa* mature biofilms after 24 h of incubation with different bacteriophages.

| Clinical isolates and ATCC strains | **Control** | **vB_PaeM_USP_1** | **vB_PaeM_USP_2** | **vB_PaeM_USP_3** | **vB_PaeM_USP_18** | **vB_PaeM_USP_25** | Total  (Strains – XTT) |
| --- | --- | --- | --- | --- | --- | --- | --- |
| Pa_Mi_1 | 0.27 (0.27) ^Aa^ | 0.48 (0.38) ^Ab^ | 0.27 (0.21) ^Aab^ | 0.23 (0.17) ^Aa^ | 0.19 (0.09) ^Aa^ | 0.23 (0.11) ^Aab^ | 0.28 (0.24) |
| Pa_Mi_2 | 0.67 (0.26) ^Bbc^ | 0.31 (0.16) ^Aab^ | 0.33 (0.12) ^Aab^ | 0.28 (0.14) ^Aa^ | 0.48 (0.40) ^Ba^ | 0.32 (0.19) ^Aab^ | 0.40 (0.26) |
| Pa_Mi_6 | 0.62 (0.33) ^Cbc^ | 0.48 (0.28) ^BCb^ | 0.36 (0.12) ^ABCab^ | 0.40 (0.09) ^ABCa^ | 0.29 (0.14) ^ABa^ | 0.19 (0.10) ^Aab^ | 0.39 (0.23) |
| Pa_Mi_7 | 0.56 (0.21) ^Babc^ | 0.22 (0.13) ^Aab^ | 0.25 (0.17) ^Aab^ | 0.20 (0.12) ^Aa^ | 0.21 (0.11) ^Aa^ | 0.20 (0.09) ^Aab^ | 0.27 (0.19) |
| Pa_Ba_164 | 0.69 (0.25) ^Bbc^ | 0.36 (0.13) ^Aab^ | 0.37 (0.18) ^Aab^ | 0.27 (0.05) ^Aa^ | 0.25 (0.15) ^Aa^ | 0.30 (0.15) ^Aab^ | 0.37 (0.21) |
| Pa_Ba_168 | 0.65 (0.36) ^Bbc^ | 0.33 (0.06) ^Aab^ | 0.35 (0.13) ^Aab^ | 0.26 (0.06) ^Aa^ | 0.22 (0.06) ^Aa^ | 0.23 (0.06) ^Aab^ | 0.34 (0.21) |
| Pa_Ba_169 | 0.40 (0.21) ^Aabc^ | 0.35 (0.11) ^Aab^ | 0.40 (0.12) ^Aab^ | 0.35 (0.16) ^Aa^ | 0.22 (0.09) ^Aa^ | 0.26 (0.13) ^aAb^ | 0.33 (0.15) |
| Pa_Trac_20 | 0.59 (0.35) ^Babc^ | 0.23 (0.11) ^Aab^ | 0.30 (0.13) ^ABab^ | 0.24 (0.19) ^Aa^ | 0.36 (0.23) ^ABa^ | 0.21 (0.19) ^Aab^ | 0.32 (0.24) |
| Pa_Trac_23 | 0.70 (0.54) ^Bbc^ | 0.29 (0.11) ^Aab^ | 0.42 (0.27) ^Ab^ | 0.25 (0.23) ^Aa^ | 0.44 (0.30) ^ABa^ | 0.46 (0.37) ^ABb^ | 0.43 (0.35) |
| Pa_Ren_1 | 0.39 (0.21) ^Aab^ | 0.25 (0.04) ^Aab^ | 0.40 (0.15) ^Aab^ | 0.32 (0.22) ^Aa^ | 0.24 (0.10) ^Aa^ | 0.32 (0.21) ^Aab^ | 0.32 (0.17) |
| Pa_ATCC 27853 | 0.44 (0.14) ^Aabc^ | 0.40 (0.28) ^Aab^ | 0.33 (0.09) ^Aab^ | 0.51 (0.36) ^Aa^ | 0.30 (0.11) ^Aa^ | 0.29 (0.08) ^Aab^ | 0.38 (0.21) |
| Pa_ATCC 2108 | 0.47 (0.48) ^Babc^ | 0.12 (0.09) ^Aa^ | 0.08 (0.02) ^Aab^ | 0.20 (0.17) ^Aba^ | 0.24 (0.34) ^ABa^ | 0.11 (0.05) ^Aa^ | 0.20 (0.28) |
| Pa_ATCC 2110 | 0.73 (0.29) ^Bc^ | 0.09 (0.06) ^Aa^ | 0.11 (0.05) ^Aa^ | 0.19 (0.15) ^Aa^ | 0.19 (0.18) ^Aa^ | 0.22 (0.17) ^Aab^ | 0.25 (0.27) |
| Pa_ATCC 2112 | 0.59 (0.25) ^Babc^ | 0.25 (0.12) ^Aab^ | 0.20 (0.08) ^Aab^ | 0.31 (0.14) ^Aba^ | 0.33 (0.38) ^ABa^ | 0.29 (0.11) ^Aab^ | 0.33 (0.23) |
| Pa_ATCC 2113 | 0.61 (0.28) ^Babc^ | 0.21 (0.11) ^Aab^ | 0.16 (0.07) ^Aab^ | 0.23 (0.12) ^Aa^ | 0.21 (0.21) ^Aa^ | 0.25 (0.19) ^Aab^ | 0.28 (0.23) |
| Total (Phages) | 0.56 (0.32) | 0.29 (0.20) | 0.29 (0.17) | 0.28 (0.18) | 0.28 (0.23) | 0.26 (0.18) |  |

Comparisons of metabolic activity were conducted be ANOVA two way with independent levels (strains and bacteriophage) and Bonferroni post-hoc test. ^ABCDE^ Different capital letters indicated statistically significant difference among bacteriophages. ^abcde^ Different small letters indicated statistically significant difference among strains.
